# Physician-modified endograft for symptomatic zone 2 penetrating ulcer of the aortic arch without bridging stenting of the left subclavian artery for vertebral preservation

**DOI:** 10.1016/j.jvscit.2024.101557

**Published:** 2024-07-02

**Authors:** Pierfrancesco Antonio Annuvolo, Ottavia Borghese, Tommaso Donati, Giovanni Tinelli, Yamume Tshomba

**Affiliations:** a Unit of Vascular Surgery, Fondazione Policlinico Universitario A. Gemelli I.R.C.C.S., Rome, Italy; b Unit of Vascular Surgery, Università Cattolica del Sacro Cuore, Rome, Italy

**Keywords:** Endovascular repair, Homemade endograft, Physician-modified stent graft, Fenestration, Surgeon-modified stent graft

## Abstract

We report the case of a 65-year-old male patient who was deemed unfit for open surgery and underwent zone 0 endovascular repair with a physician-modified fenestrated endograft for a symptomatic penetrating ulcer. A thoracic stent graft was modified creating a large fenestration for the innominate artery and the left common carotid artery, and a second small fenestration for the left subclavian artery and the left vertebral artery, which had a common origin. No bridging stent was used for the left subclavian artery to avoid coverage of the left vertebral artery. The postoperative course was uneventful, and no leaks nor other complications were detected on postoperative computed tomography angiography. Although long-term durability needs to be better assessed, our experience suggests that physician-modified fenestrated endografts are a feasible option for the emergent treatment of aortic arch lesions in unfit patients and provide satisfactory results in the short term.

Aortic arch diseases continue to be a major challenge in cardiovascular surgery and open surgical repair is still the gold standard for treatment of both aneurysms and dissections.^1^ To minimize the perioperative risks and complications associated with these complex procedures, total endovascular techniques have recently emerged as less invasive option that allow treatment also for unfit patients. Among those options, custom-made solutions seems to ensure the best technical and clinical results; however, the long waiting time required for manufacturing and the high cost remain the main constraints. Therefore, physician-modified thoracic stent grafts with homemade fenestrations, especially for aortic lesions requiring a single fenestration in zone 2, may represent an alternative option in emergent cases.^2^^,^^3^

Herein, we report the case of a total endovascular repair of a symptomatic zone 2 penetrating aortic arch ulcer using a physician-modified fenestrated endograft (PMEG) in a high-risk patient unfit for open surgery showing that endovascular treatment of aortic arch pathologies represents a valuable and effective option in emergent cases.

## Case report

A 65-year-old male patient with a history of active smoking and chronic obstructive pulmonary disease with severe resting hypoxemia, chronic ischemic cardiopathy with decreased myocardial reserve, nondisabling stroke, hypertension, and type 2 diabetes presented to our hospital with severe chest pain for 2 days in December 2023. Informed consent was obtained from the patient for publication of this Case report and any accompanying images.

**Fig 1:**
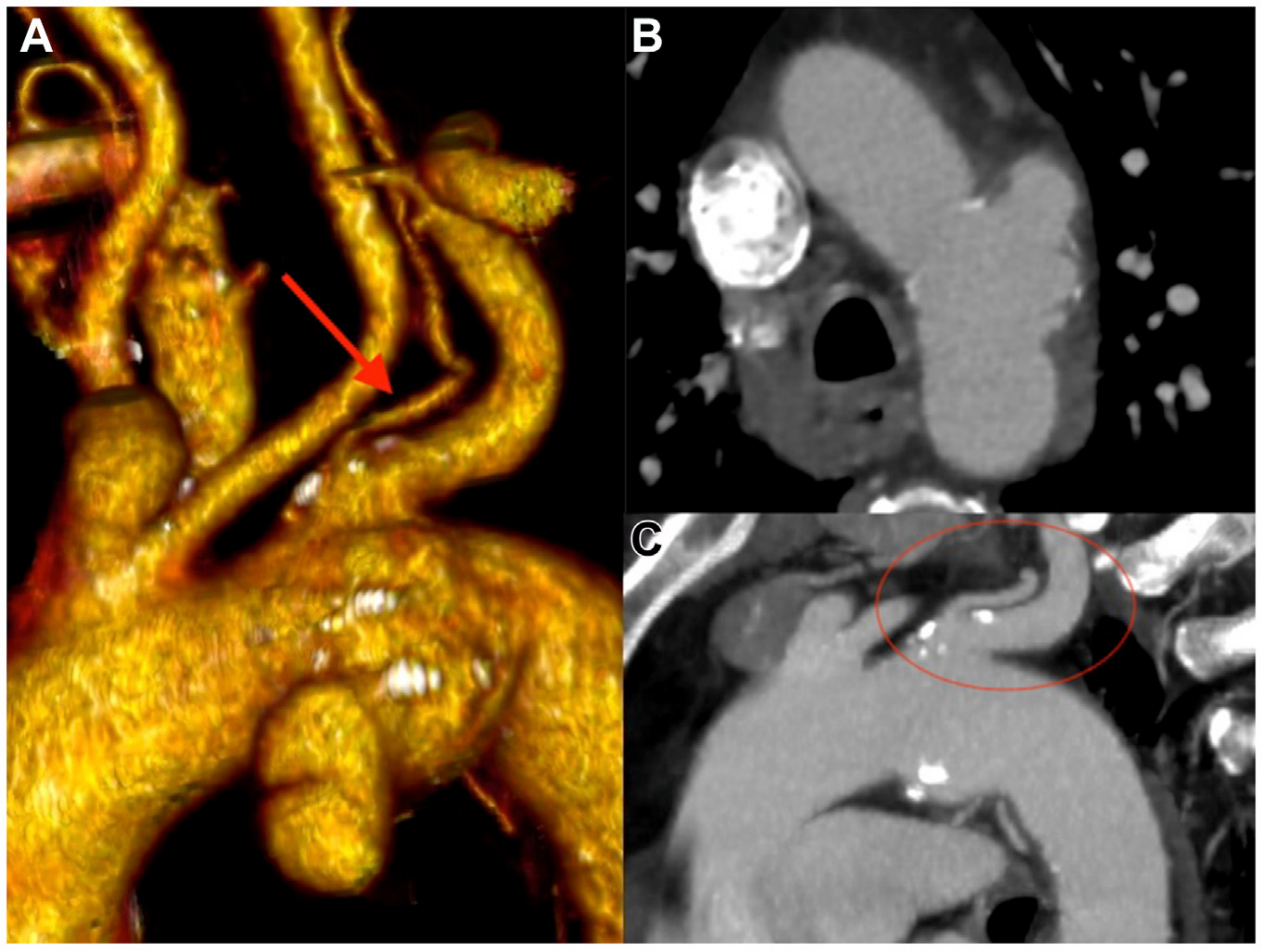
**(A-C)** Computed tomography angiography (CTA) showing the zone 2 penetrating aortic ulcer of the arch. The left vertebral artery (LVA) and its origin are highlighted (**A,** red arrow; **C,** red circle).

Urgent computed tomography angiography (CTA) revealed a penetrating aortic ulcer in Ishimaru zone 2 with a maximum transverse diameter of 35 mm and a common origin of the left subclavian artery (LSA) and the left vertebral artery (LVA) was also detected (Fig 1). Coronary artery diseases were also investigated and excluded by coronary angiography. A brain CTA was eventually performed documenting a posterior inferior cerebellar artery originating from the LVA.

Despite the optimization of medical treatment (with opioid and blood pressure target of <120/80 mm Hg), the patient was complaining persistent chest pain. A repeat CTA 48 hours after the admission was also performed showing a minimal increase in transverse diameter (37 mm) and pleural effusion. Hence, a surgical operation was scheduled. Because of his comorbidities and the supplemental long-term oxygen therapy, he was deemed unfit for open repair and an endovascular approach was, therefore, selected.

**Fig 2:**
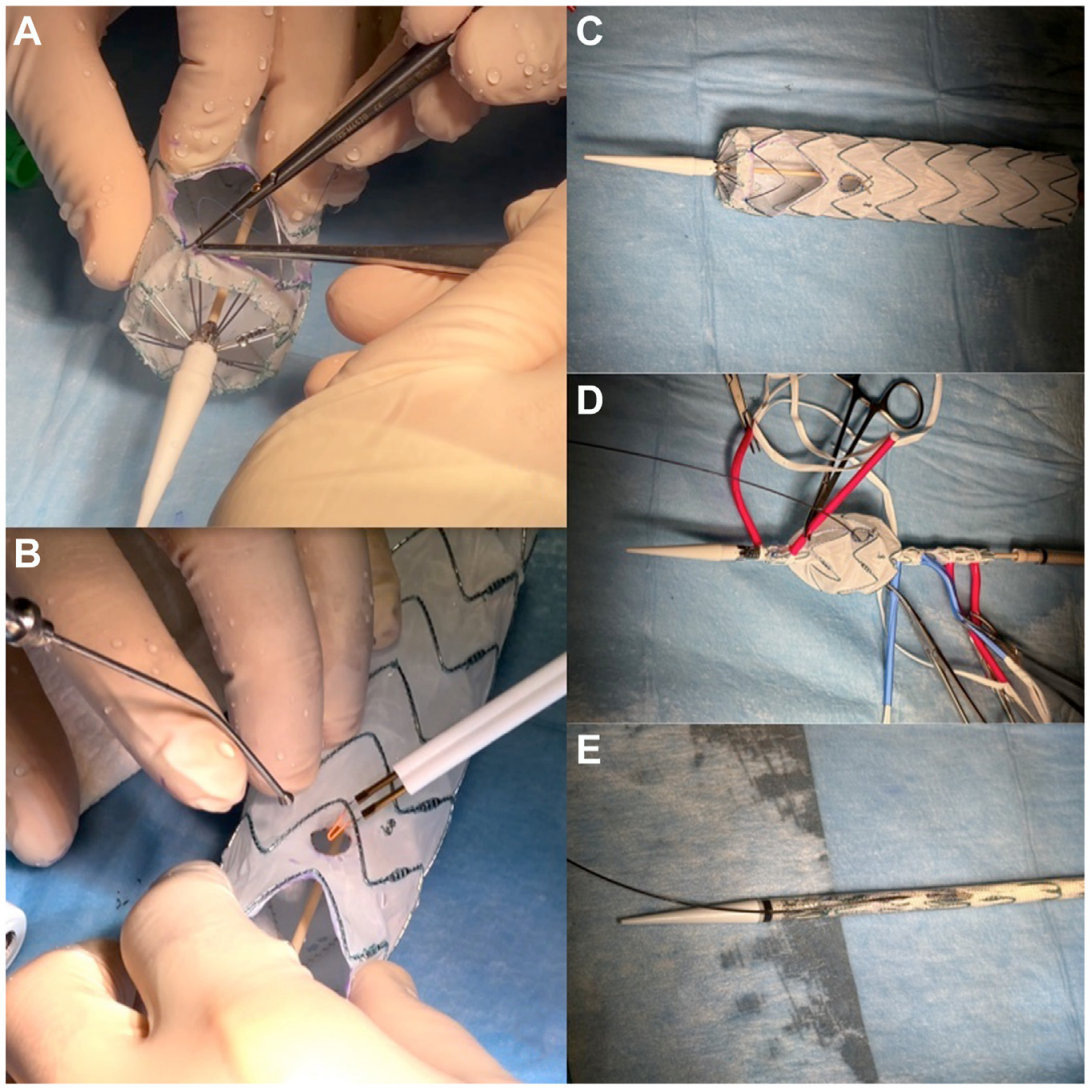
**(A)** The Medtronic Valiant Captivia thoracic stent graft was modified by first creating a large fenestration for the innominate and the left common carotid arteries. **(B)** A second smaller fenestration was created for the left subclavian artery (LSA) and vertebral artery. **(C)** The LSA fenestration was reinforced and pre-cannulated and **(D** and **E)** then the graft resheathed.

Because the patient was symptomatic, the use of a custom-made solutions was dismissed because of the long waiting time required for manufacturing and a PMEG was planned. Preoperative imaging was used to determine the size and position of the fenestrations (Fig 2, *A* and *B*).

A thoracic stent graft (Valiant Captivia 36-36-150, Medtronic Inc., Minneapolis, MN) was unsheathed partially on the table and then modified (Supplementary Video 1, online only) by first creating a large fenestration for the innominate artery and the left common carotid artery. Because of the risk of LVA coverage associated with the use of an LSA bridging stent, it was decided to create a second smaller fenestration for the revascularization of both the LSA and the LVA. The small fenestration was created using electrocautery to ensure a more precise cut; we used a scalpel for creating the larger fenestration, which was then reinforced with 5-0 polypropylene sutures near the stent to prevent the fabric from tearing.

The LSA fenestration was then reinforced suturing the tip of an extra-stiff guidewire (Lunderquist, Cook Medical LLC, Bloomington, IN) with a 5-0 polypropylene suture in a running fashion and pre-cannulated with a hydrophilic 0.035 guidewire (Fig 2, *C*). The physician-modified stent graft (PMSG) was then reloaded in its sheath using a temporary vessel loop to collapse each stent progressively (Fig 2, *D* and *E*).

Through a bilateral common femoral artery percutaneous approach, the PMSG was introduced in the aorta and positioned in the aortic arch. Digital subtraction angiography was performed to confirm the correct alignment between the PMSG radiopaque marker for the LSA and the origin of the vessel. Percutaneous left brachial artery access was obtained.

The preloaded guidewire in the LSA fenestration was used to access the LSA. A 9F introducer sheath (Flexor, Cook Medical LLC) was inserted over the LSA fenestration from the femoral access and snared out using the through-and-through wire technique. Hypotension was induced pharmacologically and the PMSG was then deployed in zone 0 (Supplementary Video 2, online only).

**Fig 3:**
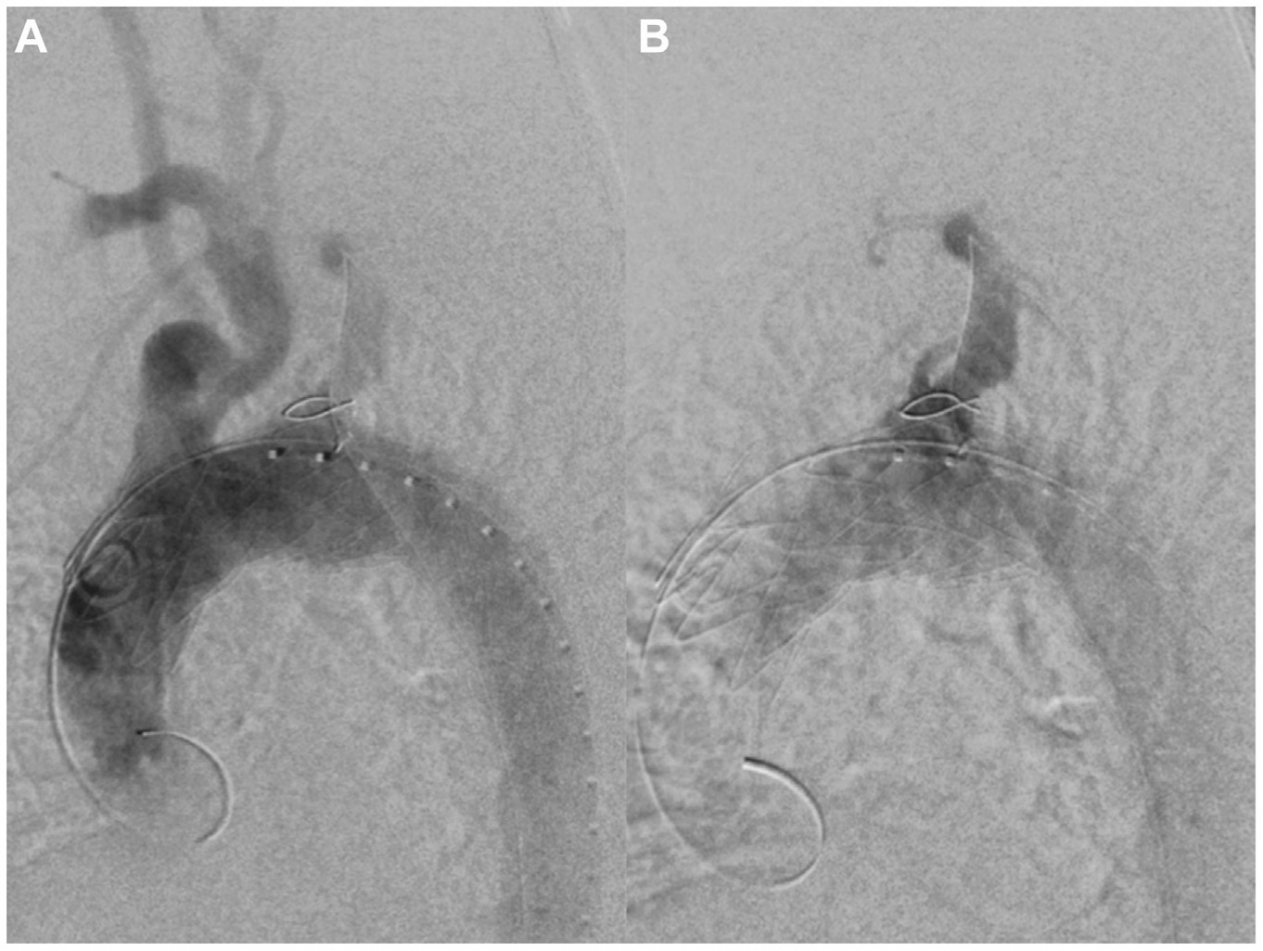
Postdeployment digital subtraction angiography showing no leaks and good perfusion of the supra-aortic vessels **(A)** and left vertebral artery (LVA) **(B)**.

Final aortic angiography showed correct positioning of the modified endograft and good LVA patency. A selective angiography through the brachial sheath was eventually performed documenting no endoleak (Fig 3).

The postoperative course was uneventful, and the patient was discharged home on postoperative day 3 in good clinical condition.

**Fig 4:**
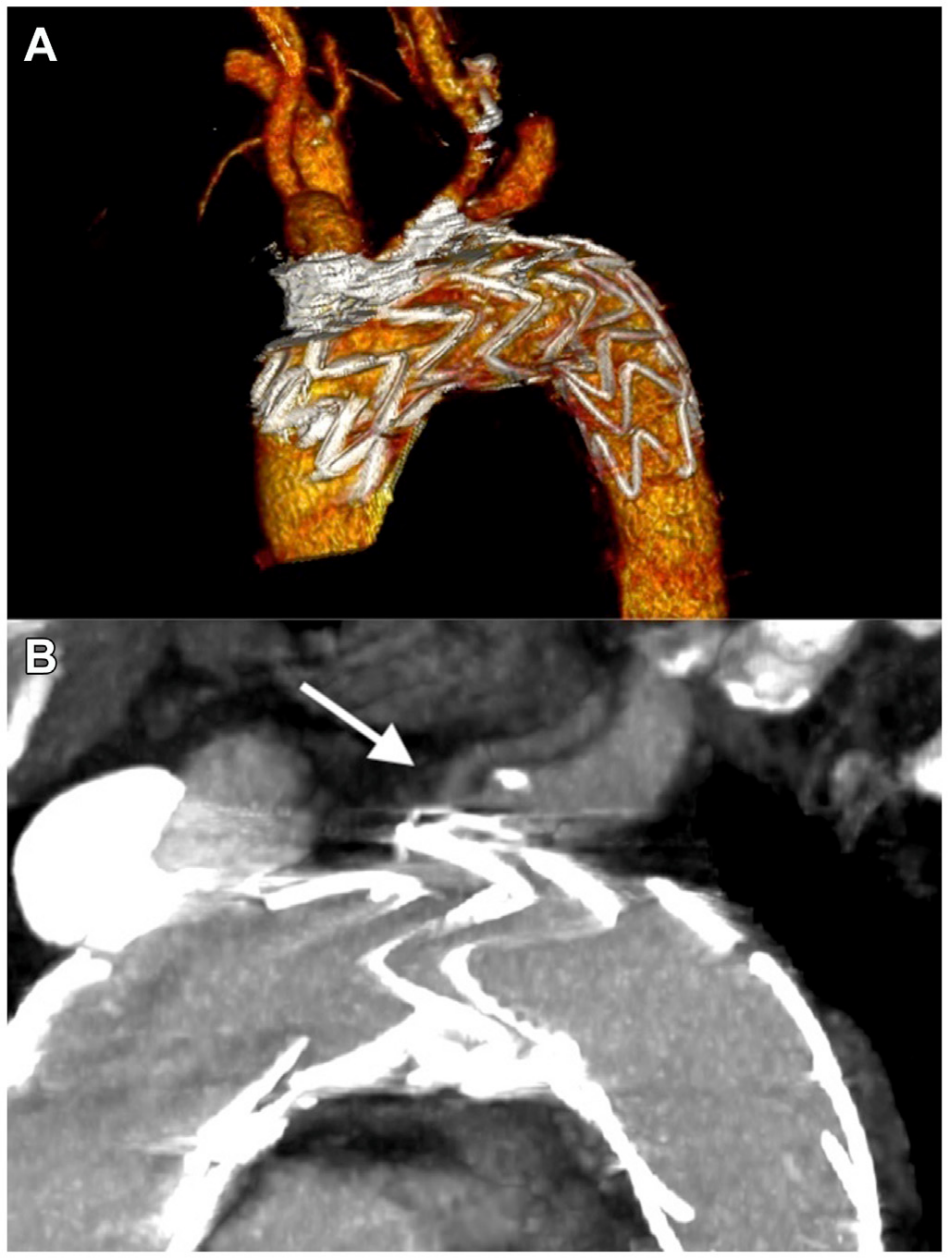
**(A)** No leaks nor other complications were detected at the postoperative computed tomography angiography (CTA). **(B)** The white arrow indicates good patency of the left vertebral artery (LVA).

A follow-up aortic CTA 3 months after surgery showed correct endograft positioning and good patency of the supra-aortic vessels including the LVA, without leaks (Fig 4). The patient is currently under ongoing clinical and imaging follow-up.

## Discussion

A variety of techniques have been described in the treatment of aortic arch pathologies,^4^, ^5^, ^6^ and despite open surgical repair is still considered the gold standard, it represents a highly invasive procedure burdened with significant perioperative and postoperative risks of complications and mortality.^7^

Hybrid techniques involving debranching of the supra-aortic vessels by extra-anatomical bypasses and thoracic endovascular aortic repair (TEVAR) endograft placement are an option when treating aortic arch pathologies with a short landing zone for a successful seal.^8^^,^^9^

In the latest years, thanks to the evolution of techniques and material, total endovascular repair has become a valuable interventional option for patients unfit for open procedures. Indeed, data from recent reports show that patients undergoing debranching TEVAR (ranging from total debranching to carotid subclavian bypass or transposition) display increased risks of morbidity and require a longer operative time and longer hospital stays when compared with patients undergoing fenestrated TEVAR.^10^, ^11^, ^12^

Custom-made devices that best fit the anatomy of the patient are currently the optimal solution for patients who are not suitable for surgery. However, as described elsewhere in this article, the cost and the long manufacturing time limit their use in emergent or urgent settings.^13^, ^14^, ^15^

Several authors have described how the use of parallel grafts (ie, chimney, periscope, snorkeler, or sandwich) could represent a valuable alternative in such conditions, but the medium- to long-term results (patency rates range between 69% and 73% of cases) are poor and the risk of endoleaks from the gutters is high (≤28% of cases).^16^^,^^17^

Conversely, previous report demonstrated that PMSG are especially a valid solution to allow revascularization of supra-aortic vessels in urgent cases.^18^, ^19^, ^20^ Specifically, single or double homemade fenestrated PMSGs have shown to be particularly effective for disease affecting Ishimaru zone 2.^21^ Indeed, data from a recent systematic review on PMEGs in treatment of several aortic arch lesions showed satisfactory early results with a 30-day mortality of 2.9% and a stroke rate of 2.1%.^22^ Additionally, the precannulation of the fenestration with a preloaded guidewire decreases neurologic risk, allowing easier cannulation of the target vessel, shortening operative time, and maximizing the technical success rate and reproducibility.^23^^,^^24^

Regarding the type of graft used for modification, despite the vast majority of data currently available is on modification of other stent graft (Zenith Alpha thoracic graft, Cook Medical), in this specific case we considered that the length of the Medtronic Valiant nasal cone may be an advantage, as well as the large distance between the peaks along the graft allow simple allocation of the fenestration that with other devices.

In this case, the urgent setting and the clinical condition of the patient did not allow the application of open, hybrid, or custom-made strategies. Moreover, the anatomical variation with a common origin of the left subclavian and left vertebral arteries increased the risk of accidental coverage or occlusion of the LVA during positioning of the bridging stent. We opted for a double homemade fenestrated stent graft, achieving good immediate technical and clinical results with no leak at the short-term follow-up. However, despite these promising outcomes, the wider applicability of PMGEs is currently limited because the long-term durability of such devices has not been investigated yet.

Although larger series have been presented in the literature, we believe the present paper adds interesting data about the feasibility and efficacy of PMEG for anatomically complex cases and in urgent setting, which has been described previously, but still requires further evaluation. At any rate, we believe that the main contribution of our work is the quality of video and imaging provided, which could be helpful for educational purposes and to allow wider knowledge and applicability of this technique.

## Conclusions

The treatment of arch pathologies represent a challenging field for the vascular surgeon. The current evolution of material and technique allow for treatment of fragile patients deemed unfit for open repair, which remains the standard of care. Custom-made devices are currently the preferred option; however, in an emergent setting PMSGs can be deployed effectively in dedicated high-volume centers. Strict follow-up is indicated, because the long-term durability of such technology has not been investigated fully.

## Funding

None.

## Disclosures

None.

## Appendix (online only)

Supplementary Video 1 (online only) A thoracic stent graft (Valiant Captivia 36-36-150, Medtronic Inc.) was unsheathed partially on table and then modified by creating fenestrations for the epiaortic vessels. The left subclavian artery (LSA) fenestration reinforcement was performed using the tip of an extra-stiff guidewire. Precannulation was done with a hydrophilic 0.035-inch guidewire. The physician-modified stent graft (PMSG) was then reloaded in its sheath using a temporary vessel loop to progressively collapse each stent.

Supplementary Video 2 (online only) The physician-modified endograft (PMEG) was positioned in the aortic arch. A 9F introducer sheath was inserted over the preloaded guidewire in the left subclavian artery (LSA) fenestration. The PMEG was then deployed in zone 0. Final angiography showed correct positioning of the modified endograft and good left vertebral artery (LVA) patency. A selective angiography through the 9F sheath was performed eventually, documenting no endoleak.

Additional material for this article may be found online at www.jvscit.org
